# Day-to-day variability in naturalistic key-hold duration is associated with self-reported Parkinson’s disease impact

**DOI:** 10.3389/fnagi.2026.1982964

**Published:** 2026-10-30

**Authors:** Yan Wang, Haoyang Hu, Jiaxuan Peng, Zekai Yu, Fei Yang

**Affiliations:** 1 Department of Neurology, The First Medical Center, Chinese PLA General Hospital, Beijing, China; 2 School of Computer Science and Technology, Hangzhou Dianzi University, Hangzhou, Zhejiang, China

**Keywords:** digital phenotyping, keystroke dynamics, naturalistic monitoring, Parkinson’s disease, within-person variability

## Abstract

**Background:**

Naturalistic keyboard use enables repeated, low-burden assessment of fine-motor behavior. Whether day-to-day variability in key-hold duration provides information beyond average key-hold duration in Parkinson’s disease (PD) remains unclear. We tested whether within-person variability in daily key-hold duration was associated with a one-time self-reported PD Impact category.

**Methods:**

We reconstructed the public Tappy dataset from raw keystroke files. The primary phenotype was the within-person coefficient of variation (CV) of daily mean key-hold duration. Ordinal-trend and categorical Impact parameterizations were treated as parallel primary analyses. Secondary and exploratory analyses evaluated alternative variability measures, timing-filter thresholds, observation density, robust daily summaries, approximate disease duration, and unequal precision of daily means. Participant-day location-scale models used both a conventional finite-keystroke sampling-variance approximation and a session/block-aware alternative; participant-cluster bootstrap inference used 2,000 replicates.

**Results:**

Quality control retained 8,999,669 keystrokes and 5,490 valid participant-days. The complete-case analysis included 73 participants with PD (33 Mild, 30 Medium, and 10 Severe). Each one-category increase in self-reported PD Impact was associated with a 32.4% higher day-to-day CV (ratio, 1.32; 95% CI, 1.13–1.55; *P* < 0.001). In the categorical parameterization, ratios were 1.38 for Medium versus Mild and 1.70 for Severe versus Mild (overall robust Wald *P* = 0.001). Estimates were essentially unchanged with 1,000- or 1,500-ms timing limits and a hold-duration-only filter. Daily-median CV remained associated with Impact (ratio, 1.30; 95% CI, 1.08–1.56; *P* = 0.005). Among the same 52 participants eligible at 500 keystrokes/day, the ratio was 1.25 using the 100-keystroke definition and 1.20 using the 500-keystroke definition. In 72 participants contributing 3,193 participant-days, the session/block-aware location-scale model estimated a 1.62-fold higher latent residual SD per Impact category (2,000-replicate participant-cluster bootstrap 95% CI, 1.20–1.98).

**Conclusion:**

Greater self-reported PD Impact was associated with higher within-person variability in naturalistic key-hold duration. This cross-sectional association identifies day-to-day variability as a candidate feature for passive fine-motor assessment. Prospective validation against repeated clinical outcomes and independent naturalistic cohorts is needed.

## Introduction

Parkinson’s disease (PD) is a progressive neurodegenerative disorder characterized by bradykinesia and impaired fine-motor control (Bloem et al., 2021). The Movement Disorder Society-sponsored Unified Parkinson’s Disease Rating Scale (MDS-UPDRS) is a reference clinical instrument, but scheduled examinations provide only episodic observations (Goetz et al., 2008). Digital measures can extend assessment into daily life, although clinical use requires evidence of validity, reliability, usability, and robustness to context (Espay et al., 2016; Maetzler et al., 2013).

Smartphone and wearable studies have established the feasibility of remote PD assessment across large cohorts and repeated follow-up (Adams et al., 2023, 2024; Bot et al., 2016; Morgan et al., 2020; Prince et al., 2018; Sahandi Far et al., 2021; Sun et al., 2024). Continuous mobility measures, motor-fluctuation monitoring, digital motor diaries, and decentralized cohorts further show that frequent measurement can capture behavior outside the clinic (Erb et al., 2020; Evers et al., 2020; Powers et al., 2021; Schneider et al., 2021; Shah et al., 2020). These studies also highlight a central measurement problem: digital features can vary with adherence, context, and observation density (Sahandi Far et al., 2021; Schneider et al., 2021).

Typing provides a low-burden view of fine-motor behavior because key-down and key-up timing can be recorded during ordinary computer use. Natural-typing studies have detected psychomotor impairment and PD-related differences in laboratory and at-home settings (Adams, 2017a; Alfalahi et al., 2022; Arroyo-Gallego et al., 2018; Giancardo et al., 2015, 2016; Liu et al., 2023; Tripathi et al., 2023). Subsequent work has examined treatment response, cross-dataset generalization, hand-specific patterns, hold-time fluctuations, and touchscreen typing (Arroyo-Gallego et al., 2017; Holmes et al., 2023; Iakovakis et al., 2018; Lan and Yeo, 2019; Matarazzo et al., 2019; Tat et al., 2025; Tripathi et al., 2024). However, most studies have emphasized case-control discrimination, aggregate features, or short-term treatment effects rather than day-to-day within-person variability.

We tested whether day-to-day variability in daily mean key-hold duration was associated with a one-time self-reported PD Impact category in the public Tappy dataset. This analysis relates longitudinal typing measurements to cross-sectional self-reported impact, which has not been validated as a clinical severity scale. We defined CV as the primary relative-variability phenotype and used RMSSD, raw within-person SD, and mean hold duration as complementary measures. We then examined alternative phenotype definitions, observation density, approximate disease duration, and unequal sampling precision of daily means.

## Materials and methods

### Study design and data source

We conducted a secondary observational analysis of the Tappy Keystroke Data resource distributed through PhysioNet (Adams, 2017a,b). The source study recruited adults described as 50–80 years old and installed a background application on Windows computers. The source application recorded keystroke timing and coarse keyboard-position information during ordinary computer use. The public release used here provides timing and keyboard-side labels, but no typed characters or keyboard-column positions (Adams, 2017a,b). Numeric keys were not recorded in the source study (Adams, 2017a). Recruitment occurred between July 2016 and March 2017. The released data contained valid records from 7 June 2016 to 4 March 2017, all of which were retained. The present study evaluated participant-level associations between a longitudinally derived typing phenotype and a one-time self-reported Parkinson’s disease (PD) Impact category. It did not assess within-person clinical progression. The original study received Charles Sturt University Human Research Ethics Committee approval (H17013) and obtained informed consent (Adams, 2017a). The current anonymized public-data analysis involved no new recruitment or contact and required no additional ethical approval.

### Keystroke processing and phenotype construction

Raw keystroke files were linked to participant metadata through de-identified user identifiers. Key-hold duration was defined as the interval from key-down to key-up for the same keystroke. Inter-key latency measured the interval between successive key-down events, whereas flight time measured the interval from the preceding key-up event to the current key-down event. All three measures were expressed in milliseconds (Adams, 2017a,b). The primary quality-control rule retained records with a valid date and key-hold duration, inter-key latency, and flight time each greater than 0 and no more than 800 ms. These limits defined the retained-event sample and were not disease-specific thresholds. Retained records were grouped by participant and calendar date. A valid participant-day required at least 100 retained keystrokes, and a participant entered the longitudinal cohort after contributing at least five valid days.

For each valid participant-day, we calculated mean key-hold duration and retained-keystroke count. Let m_ij_ denote the daily mean for participant i on valid day j, and let D_i_ denote the number of valid days. The primary phenotype was the within-person coefficient of variation (CV), defined as:


$$C\,V_{\text{i}}=S\,D\,\left(m_{\text{ij}}\right)/m\,e\,a\,n\,\left(m_{\text{ij}}\right).$$

We also calculated the unnormalized within-person SD to determine whether an association with CV could be attributed to its mean denominator. Temporal instability was summarized using the root mean square of successive differences (RMSSD):


$${\mathrm{RMSSD}}_{\text{i}}={\left[\Sigma_{j=1}^{\mathrm{Di}-1}\,{\left(m_{i,j+1}-m_{\mathrm{ij}}\right)}^{2}/\left(D_{i}-1\right)\right]}^{1/2}.$$

Additional phenotype definitions were used to assess sensitivity to within-day skew and isolated extreme observations. These analyses replaced each daily mean with the corresponding daily median or 10% trimmed mean before recalculating participant-level variability. An exploratory variant restricted RMSSD to consecutive calendar days. For the exploratory hand-standardized analysis, L and R labels represented keyboard-side classifications rather than observed hand use. On valid days with at least 25 retained keystrokes bearing each label, the side-specific mean key-hold durations were averaged with equal weights. Participant-level CV was calculated across these standardized daily means, requiring at least five eligible days. Records without an L or R label did not contribute to the side-specific means.

### Exposure, covariates, and analytic population

The analytic population comprised participants who reported PD and had a non-missing public Impact category. The PhysioNet release describes Impact as PD severity or impact on daily life and provides three self-reported response options: Mild, Medium, and Severe (Adams, 2017b). The original question, administration instructions, and validation evidence are unavailable in the release. Impact was assessed once and analyzed as a self-reported category (Supplementary Note 1).

Impact was represented in two complementary ways. The ordinal parameterization coded Mild, Medium, and Severe as 1, 2, and 3, respectively. The categorical parameterization used Mild as the reference and did not assume equal spacing between categories. Participant metadata also provided sex, birth year, DiagnosisYear, tremor status, and reported use of levodopa, dopamine agonists, and monoamine oxidase B inhibitors. Age was estimated as 2016.5 minus birth year. Approximate disease duration was calculated as the year of each participant’s observation midpoint minus DiagnosisYear and was truncated at zero. This approximation reflected the year-level precision of DiagnosisYear. Observation density was represented by the number of valid typing days and the median number of retained keystrokes per valid day.

The primary analysis used complete cases for Impact, age, sex, observation-density covariates, and participant mean hold duration. This population included 73 participants with PD. Missing values were not imputed. An age-omitted analysis retained all 87 participants with known Impact and was used to assess the influence of complete-case selection.

### Primary and complementary participant-level analyses

The primary outcome was the natural logarithm of the participant-level CV. The ordinal-trend model included Impact coded from 1 to 3, age, sex, log-transformed number of valid typing days, log-transformed median daily keystroke count, and log-transformed participant mean hold duration. The categorical model used the same covariates and estimated Medium-versus-Mild and Severe-versus-Mild contrasts. Overall evidence for the categorical exposure was evaluated using a joint two-degree-of-freedom Wald test. The ordinal and categorical parameterizations addressed the same primary question and were interpreted in parallel.

Participant-level models were fitted by ordinary least squares with HC3 heteroskedasticity-consistent standard errors. Coefficients from models with log-transformed outcomes were exponentiated and reported as multiplicative ratios with 95% confidence intervals. Complementary models examined RMSSD, within-person SD, and participant mean hold duration. The within-person SD model omitted participant mean hold duration to avoid denominator-related coupling. An analogous RMSSD model without adjustment for mean hold duration was also evaluated. All model-based *P*-values were two-sided. Secondary and exploratory analyses were interpreted using effect estimates, confidence intervals, and concordance with the primary analysis, rather than as independent discoveries.

*Post hoc* analyses evaluated CV across daily mean inter-key latency and flight time in the fixed 73-participant primary cohort, using the same retained keystrokes and valid-day rules. Ordinal and categorical models used the primary covariate framework, replacing mean key-hold duration with the participant mean of the corresponding interval. Holm adjustment covered four tests, comprising two ordinal trends and two categorical omnibus tests. Categorical contrast *P*-values and pointwise confidence intervals were unadjusted.

### Participant-day location-scale analysis

We used participant-day location-scale models to evaluate whether unequal numbers of valid days and retained keystrokes affected the estimated association with variability. These exploratory analyses included 72 complete-case participants with a recorded DiagnosisYear. Approximate disease duration was first added to the participant-level CV, SD, and RMSSD models. We then analyzed the 3,193 participant-day observations directly.

For participant i on day j, the location model was:


$$y_{i\,j}=x_{i}^{T}\,\beta +b_{i}+\varepsilon_{i\,j},$$

where y_ij_ was log daily mean hold duration and b_*i*_ was a Gaussian participant random intercept with variance τ^2^. Latent day-to-day residual variance followed a log-linear scale model:


$$\log\,\left(\sigma_{i}^{2}\right)=z_{i}^{T}\,\gamma .$$

Both x_i_ and z_i_ included Impact, age per 10 years, sex, approximate disease duration per 5 years, log-transformed number of valid typing days, and log-transformed median daily keystroke count. The Impact coefficient in the scale equation was exponentiated as exp(γImpact/2) and interpreted as the ratio of latent residual SD per one-category increase in self-reported PD Impact.

Three variance specifications were evaluated. The first model did not include a daily sampling-variance term. The conventional measurement-error model specified:


$$\mathrm{Var}\,\left(\varepsilon_{\mathrm{ij}}\right)=\sigma_{i}^{2}+v_{\mathrm{ij}},$$

where v_ij_ was approximated by the within-day key-hold duration variance divided by the retained-keystroke count and squared daily mean. This delta-method approximation treats retained keystrokes within a participant-day as conditionally independent.

The session/block-aware model replaced this approximation with a cluster-sandwich estimate of the variance of each daily mean. Typing sessions were separated by gaps longer than 30 min. When only one session was identifiable, the participant-day was divided into consecutive blocks of 50 keystrokes. The resulting variance of the daily mean was divided by squared daily mean hold duration for analysis on the log scale. These operational session and block definitions addressed within-day dependence but did not identify unrecorded typing tasks.

Location-scale models were fitted by marginal maximum likelihood with bounded variance parameters. Optimization used L-BFGS-B with ftol = 10^−10^, gtol = 10^−7^, maxls = 40, and a maximum of 600 iterations. Each model estimated 15 parameters. Convergence status, boundary behavior, maximum absolute gradients, likelihood values, and failed bootstrap fits were retained as diagnostic outputs.

### Robustness analyses and computational reproducibility

Robustness analyses examined four potential sources of instability. First, we repeated the analysis with the upper inclusion bound increased from 800 to 1,000 or 1,500 ms for each of the three timing measures, retaining the requirement that each value be greater than zero. A separate sensitivity analysis applied the interval criterion of >0 to ≤800 ms only to key-hold duration. Second, observation-window analyses recalculated participant-level phenotypes within the first 14, 30, 60, 90, or 120 calendar days after each participant’s first valid day. Fixed-day analyses used exactly the first 5, 10, 20, or 30 valid typing days and required participants to contribute the corresponding number of days. Third, temporal influence was assessed by omitting each calendar month and rebuilding all participant-level phenotypes. Fourth, data-density analyses varied the minimum numbers of valid days and keystrokes per day. The most stringent comparison fixed the cohort to the 52 complete cases eligible at 500 keystrokes per day and recalculated CV using both the 100- and 500-keystroke definitions.

Further analyses omitted age, restricted estimated age to 50–80 years, adjusted for observation span, and added approximate disease duration, medication use, and tremor. Medication and tremor were treated as clinical-characteristic sensitivity covariates, rather than as comprehensive adjustment for PD clinical severity or contemporaneous medication state. A centered quadratic Impact term provided a limited assessment of departure from linearity. Its interpretation was constrained by the small number of participants in the Severe group.

Participant-level uncertainty was evaluated using 10,000 bootstrap samples drawn with replacement within Impact category from the fixed complete-case population. The primary CV and RMSSD models were refitted in every sample. Location-scale models used 2,000 participant-cluster bootstrap replicates. Measurement-error-aware models additionally used 2,000 Mammen participant-cluster wild-bootstrap replicates. Fixed random seeds were used throughout. Failed fits were retried from the full-sample solution, and all failure counts were recorded.

Data processing and statistical analyses were performed in Python 3.12.4 using NumPy 2.2.6, pandas 2.2.3, SciPy 1.14.1, statsmodels 0.14.4, and Numba 0.61.2. Figures were generated using Matplotlib. The workflow was rerun from the archived Tappy user and keystroke files supplied with PhysioNet v1.0.0.

## Results

### Cohort construction and observation density

The Tappy archive contained 9,014,871 raw keystroke records linked to participant metadata. Quality control retained 8,999,669 records and produced 5,490 participant-days with at least 100 valid keystrokes. Requiring at least five valid days yielded 121 participants (89 with self-reported PD and 32 controls). Among participants with PD, 87 reported Mild, Medium, or Severe Impact (37, 38, and 12 participants, respectively). Fourteen lacked estimated age, leaving 73 complete cases: 33 Mild, 30 Medium, and 10 Severe (Figure 1 and Table 1). DiagnosisYear was available for 72 complete cases, who contributed 3,193 participant-days to the disease-duration and location-scale analyses.

**FIGURE 1:**
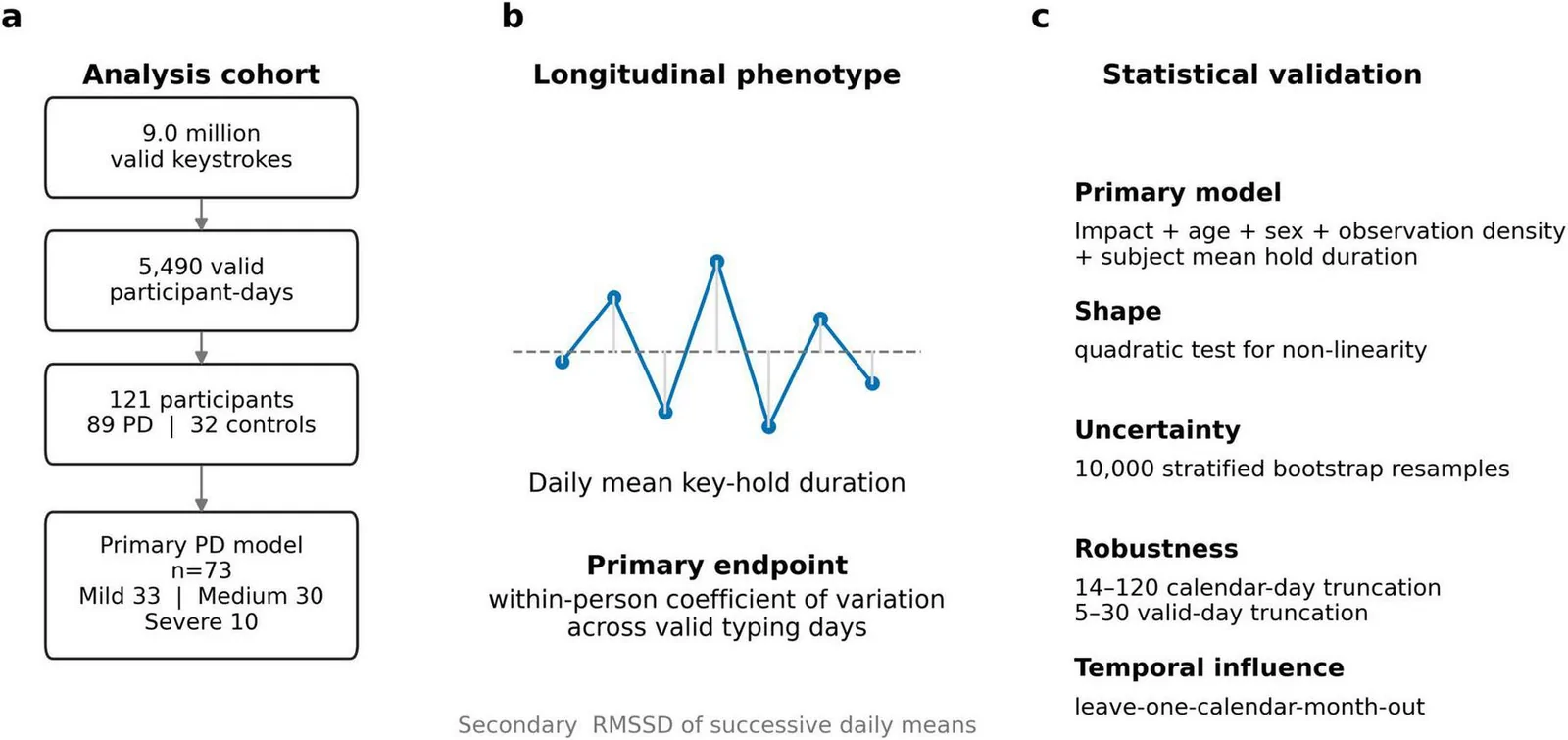
Study design and analytic framework. **(a)** Keystroke-level quality control, daily aggregation, and construction of the longitudinal and complete-case PD cohorts. Controls were retained in the data-flow accounting but were not part of the study hypothesis or association analyses. **(b)** Daily mean key-hold duration was calculated for each valid typing day; the primary phenotype was the within-person coefficient of variation across days, with RMSSD of successive valid-day means as a complementary absolute instability measure. **(c)** The adjusted association with self-reported PD Impact category was evaluated using ordinal and categorical parameterizations, participant-level resampling, observation-window truncation, data-density sensitivity, and temporal influence analyses. PD, Parkinson’s disease; RMSSD, root mean square of successive differences.

**TABLE 1:** Characteristics of the primary complete-case Parkinson’s disease cohort.

Characteristic	Mild self-reported PD Impact	Medium self-reported PD Impact	Severe self-reported PD Impact
Participants, *n*	33	30	10
Estimated age, years	67.3 (9.4)	65.9 (9.0)	68.8 (6.9)
Male sex, *n* (%)	11 (33.3)	15 (50.0)	4 (40.0)
Tremor reported, *n* (%)	22 (66.7)	19 (63.3)	6 (60.0)
Levodopa use, *n* (%)	15 (45.5)	21 (70.0)	8 (80.0)
Dopamine agonist use, *n* (%)	5 (15.2)	6 (20.0)	5 (50.0)
Monoamine oxidase B inhibitor use, *n* (%)	2 (6.1)	1 (3.3)	1 (10.0)
Valid typing days	30 (16–66)	16.5 (9–37.3)	30 (12.8–41.3)
Median valid keystrokes per day	646 (278–1,054)	404 (258–848)	743 (337–1,468)
Observation span, days	50 (24–132)	33.5 (17.8–75.8)	62.5 (32.5–82.3)
Mean hold duration, ms	120.6 (107.6–145.3)	113.6 (95.5–146.9)	132.2 (101.5–151.0)
Day-to-day key-hold duration CV	0.055 (0.043–0.070)	0.082 (0.055–0.105)	0.091 (0.055–0.140)
Key-hold duration RMSSD, ms	7.87 (6.29–11.27)	12.10 (7.32–17.39)	16.54 (6.62–32.27)

Data are mean (standard deviation), *n* (%), or median (interquartile range). Age was approximated at the midpoint of the original data-collection period from reported birth year. CV, coefficient of variation; RMSSD, root mean square of successive differences.

### Higher self-reported PD impact was associated with greater day-to-day variability in key-hold duration

Day-to-day CV increased across self-reported Impact categories, with medians of 0.055 for Mild, 0.082 for Medium, and 0.091 for Severe Impact (Figure 2a). In the adjusted ordinal model, each one-category increase in Impact was associated with 32.4% higher CV (ratio, 1.32; 95% CI, 1.13–1.55; *P* < 0.001; Figure 2b). The categorical model also supported an overall difference (robust Wald *P* = 0.001). Ratios were 1.38 for Medium versus Mild (95% CI, 1.09–1.75; *P* = 0.007) and 1.70 for Severe versus Mild (95% CI, 1.18–2.45; *P* = 0.004). Thus, the primary result did not depend solely on treating the three categories as equally spaced. The primary ordinal and categorical estimates are summarized in Table 2, with detailed categorical results provided in Supplementary Table 4.

**FIGURE 2:**
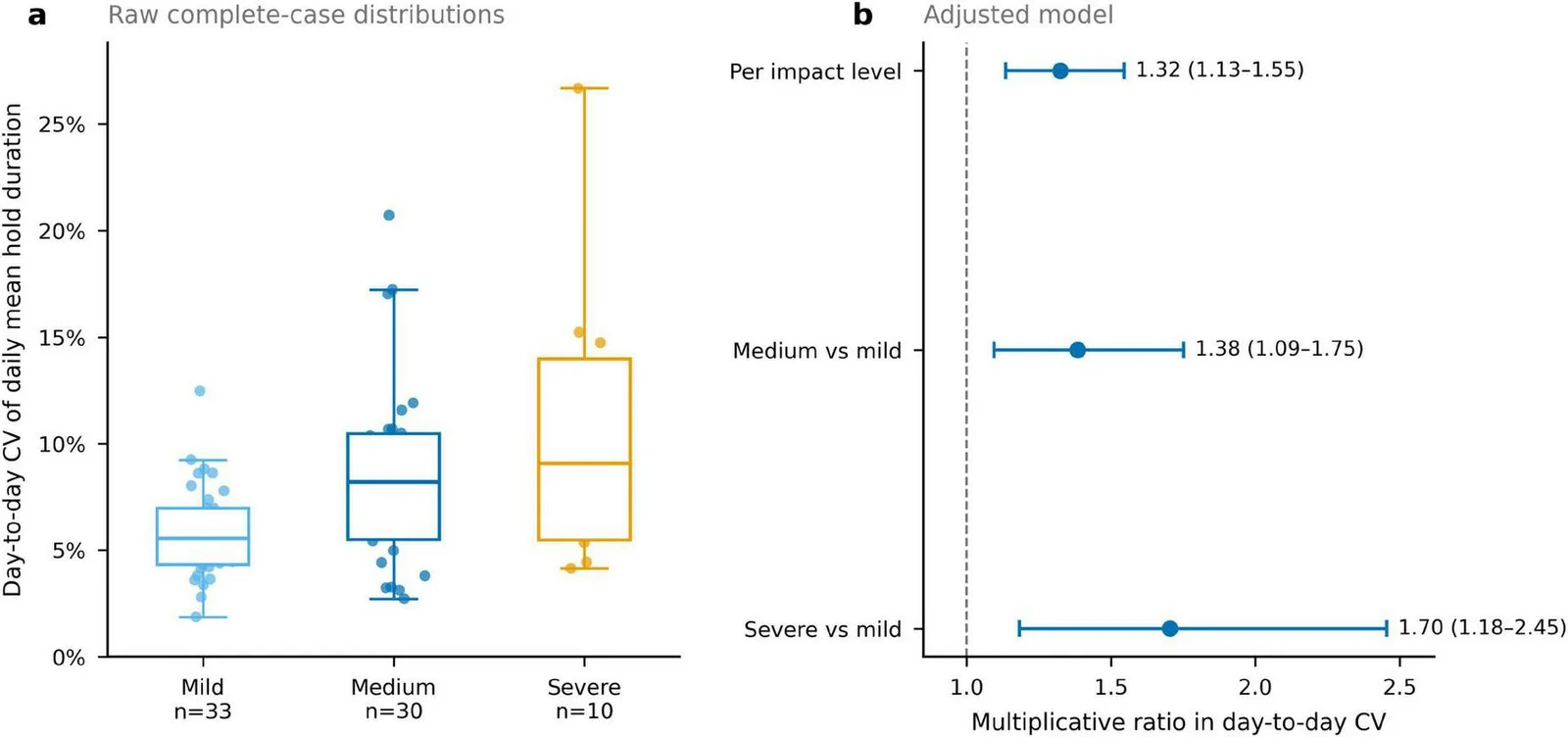
Self-reported PD Impact gradient in day-to-day variability in key-hold duration. **(a)** Raw complete-case distributions of the within-person CV of daily mean key-hold duration among participants with Mild (*n* = 33), Medium (*n* = 30), or Severe (*n* = 10) self-reported PD Impact. Boxes show medians and interquartile ranges; points are individual participants. **(b)** Adjusted multiplicative ratios and 95% confidence intervals from the parallel ordinal-trend and categorical parameterizations, using HC3 heteroskedasticity-consistent standard errors. Models adjusted for age, sex, valid typing days, median daily typing volume, and participant mean hold duration. CV, coefficient of variation; PD, Parkinson’s disease.

**TABLE 2:** Primary association and key supporting analyses.

Analysis	Ratio	95% confidence interval	*P*-value
Ordinal trend in day-to-day CV, per Impact category	1.32	1.13–1.55	<0.001
Categorical Impact overall (2 df)	–	–	0.001
Medium versus Mild day-to-day CV	1.38	1.09–1.75	0.007
Severe versus Mild day-to-day CV	1.70	1.18–2.45	0.004
RMSSD, per Impact category	1.36	1.15–1.61	<0.001
Raw within-person SD, no mean adjustment	1.39	1.09–1.76	0.007
Consecutive-calendar-day RMSSD	1.40	1.12–1.74	0.003
50/50 hand-standardized CV	1.33	1.15–1.53	<0.001
Mean hold duration, per Impact category	1.03	0.93–1.14	0.515
Clinical-characteristic sensitivity: medication and tremor covariates	1.29	1.07–1.56	0.009
10,000-bootstrap primary estimate	1.32	1.15–1.53	–
CV + approximate disease duration	1.34	1.14–1.57	<0.001
ME-aware participant-day residual SD	1.58	1.13–1.91	–
Session/block-aware participant-day residual SD	1.62	1.20–1.98	–

Participant-level ratios are exponentiated coefficients from log-scale models using HC3 standard errors. The disease-duration row adds approximate disease duration and contains 72 participants. Participant-day rows report latent residual SD ratios from measurement-error-aware heteroskedastic random-intercept location-scale models (72 participants; 3,193 participant-days). Confidence intervals are percentile intervals from 2,000 participant-cluster bootstrap resamples. The session/block-aware model estimates daily sampling variance using detected sessions or consecutive 50-keystroke blocks. CV, coefficient of variation; ME, measurement error; RMSSD, root mean square of successive differences; SD, standard deviation. Ratios and confidence intervals are displayed to two decimal places; unrounded estimates are available from the corresponding author upon reasonable request.

### Complementary instability and mean key-hold duration analyses

Complementary absolute-variability measures supported the primary result. Median RMSSD increased from 7.87 ms in Mild to 12.10 ms in Medium and 16.54 ms in Severe Impact. The adjusted ratio per category was 1.36 (95% CI, 1.15–1.61; *P* < 0.001). Raw within-person SD, modeled without mean hold duration, was also associated with Impact (ratio, 1.39; 95% CI, 1.09–1.76; *P* = 0.007). Consecutive-calendar-day RMSSD and hand-standardized CV produced similar estimates. In contrast, mean hold duration was not associated with Impact (ratio, 1.03; 95% CI, 0.93–1.14; *P* = 0.515). Additional phenotype results are reported in Supplementary Table 3.

### Exploratory inter-key variability analyses

In the fixed primary cohort, the ordinal CV ratio per self-reported PD Impact category was 1.20 for inter-key latency (95% CI, 0.99–1.46; *P* = 0.062; Holm-adjusted *P* = 0.124) and 1.18 for flight time (95% CI, 1.01–1.38; *P* = 0.039; Holm-adjusted *P* = 0.118). All four Holm-adjusted *P*-values exceeded 0.05 (Supplementary Table 9).

### Disease-duration and participant-day location-scale analyses

DiagnosisYear was available for 72 complete cases. Adding approximate disease duration produced a CV ratio of 1.34 per Impact category (95% CI, 1.14–1.57; *P* < 0.001). Associations also persisted for raw within-person SD (ratio, 1.40; 95% CI, 1.10–1.79; *P* = 0.006) and RMSSD (ratio, 1.46; 95% CI, 1.13–1.88; *P* = 0.004).

Exploratory direct modeling of 3,193 participant-days produced directionally concordant estimates. Each one-category increase in Impact was associated with a 1.53-fold higher latent residual SD in the location-scale model (2,000-replicate participant-cluster bootstrap 95% CI, 1.13–1.81). Incorporating conventional finite-keystroke sampling variance yielded a residual SD ratio of 1.58 (participant-cluster bootstrap 95% CI, 1.13–1.91; Mammen cluster wild-bootstrap 95% CI, 1.16–2.17). The session/block-aware sampling-variance model yielded a residual SD ratio of 1.62 (participant-cluster bootstrap 95% CI, 1.20–1.98; Mammen cluster wild-bootstrap 95% CI, 1.14–2.27). The median session/block-aware sampling variance was 2.26 times the conventional approximation. All full-sample fits converged; none of the 2,000 replicates failed in any reported bootstrap. Approximate disease duration was not associated with latent residual SD in these models (Supplementary Tables 6, 8).

### Shape and resampling analyses

The exploratory quadratic term did not indicate detectable curvature, although the Severe group was too small to support a flexible dose-response assessment. A 10,000-replicate stratified participant bootstrap yielded a median primary ratio of 1.32 and a percentile 95% interval of 1.15–1.53. Leave-one-participant-out estimates ranged from 1.28 to 1.38, and all model-based 95% confidence intervals remained above 1 (Supplementary Table 3).

### Observation-window and temporal robustness

Estimates were directionally consistent across most observation-window, influence, filtering, and alternative-phenotype analyses. Longer fixed-day requirements reduced the eligible sample and widened confidence intervals. In the same 52 participants, increasing the daily threshold from 100 to 500 keystrokes attenuated the ratio from 1.25 to 1.20, with the latter interval crossing 1. Phenotype definition and the observation pattern therefore affected the estimate and its precision. Calendar-window and exact fixed-day analyses are summarized in Figure 3 and Supplementary Table 1. Leave-one-calendar-month-out estimates were stable (Supplementary Figure 1 and Supplementary Table 2).

**FIGURE 3:**
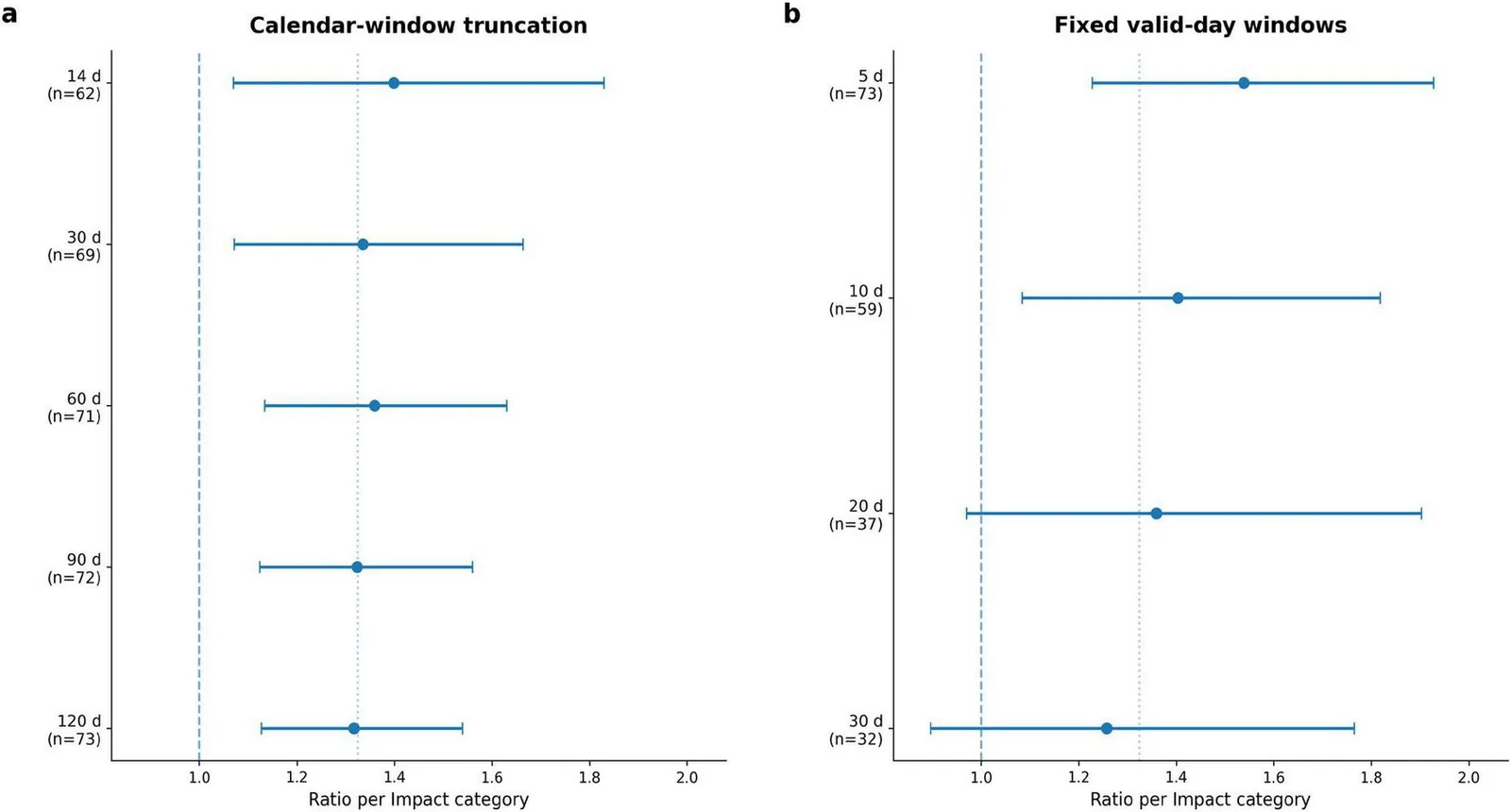
Robustness to observation-length restriction. **(a)** The within-person day-to-day CV was recomputed using typing observations within the first 14, 30, 60, 90, or 120 calendar days after each participant’s first valid day. **(b)** Exact fixed-day analyses used the first 5, 10, 20, or 30 valid typing days and required participants to have at least that number of valid days; sample sizes are shown in the labels. Points show adjusted multiplicative ratios per one-category increase in self-reported PD Impact; error bars are 95% confidence intervals from HC3 robust standard errors. The dashed line denotes the null ratio of 1 and the dotted line denotes the primary full-observation estimate (1.32). CV, coefficient of variation; PD, Parkinson’s disease.

### Covariate, sample, and data-density sensitivity analyses

The clinical-characteristic sensitivity analysis that added reported medication use and tremor produced a similar estimate, but these variables do not constitute comprehensive adjustment for PD clinical severity or time-varying medication state. Omitting age expanded the sample to all 87 participants with known Impact and yielded a ratio of 1.31 (95% CI, 1.14–1.50; *P* < 0.001). No large descriptive differences were observed between the 73 age-complete participants and the 14 with missing age, although the small missing-age group precludes an assumption of ignorable missingness (Supplementary Table 5). Timing-filter sensitivity analyses were essentially unchanged: ratios were 1.32 with 1,000- and 1,500-ms upper limits and with the hold-duration-only 0-800-ms rule. Daily-median CV (ratio, 1.30; 95% CI, 1.08–1.56; *P* = 0.005), daily-median RMSSD (ratio, 1.35; 95% CI, 1.11–1.63; *P* = 0.002), and 10% trimmed-mean CV (ratio, 1.32; 95% CI, 1.12–1.55; *P* < 0.001) were concordant. In the fixed 52-participant cohort eligible at 500 keystrokes/day, the ratio was 1.25 using the 100-keystroke definition (95% CI, 1.04–1.50; *P* = 0.020) and 1.20 using the 500-keystroke definition (95% CI, 0.97–1.49; *P* = 0.088), indicating that both phenotype definition and reduced precision may contribute to attenuation. These filtering, robust daily-summary, and same-sample analyses are detailed in Supplementary Table 7.

## Discussion

Higher self-reported PD Impact was associated with greater day-to-day variability in naturalistic key-hold duration. The association was evident for relative and absolute variability measures, including daily-median and trimmed-mean variants, but not for average hold duration. Timing-filter thresholds had negligible influence. Adjustment for approximate disease duration and participant-day models that used either conventional or session/block-aware daily sampling variance produced concordant estimates.

Previous typing studies have focused mainly on PD-control discrimination, aggregated motor indices, and treatment response (Adams, 2017a; Alfalahi et al., 2022; Arroyo-Gallego et al., 2018; Giancardo et al., 2016; Liu et al., 2023; Matarazzo et al., 2019; Tripathi et al., 2023, 2024). The present analysis examined variability across days after accounting for average key-hold duration and observation density.

Coefficient of variation is mathematically coupled to its denominator, yet unnormalized within-person SD remained associated with Impact without mean adjustment. RMSSD was also concordant with and without mean adjustment. Consecutive-calendar-day analyses reduced sensitivity to irregular temporal gaps, and equal weighting of keyboard-side means reduced sensitivity to changing proportions of left- and right-side keystrokes.

The *post hoc* inter-key analyses yielded Holm-adjusted *P*-values above 0.05, with confidence intervals compatible with a range of associations. The clinical relevance of latency and flight-time variability requires independent validation.

Participant-day models that incorporated daily sampling variance yielded associations consistent with the participant-level results. The session/block-aware variance estimate exceeded the conventional estimate, while the estimated Impact association remained positive. Unrecorded typing tasks, serial dependence beyond the chosen blocks, and time-varying medication state may still contribute to residual day-to-day variability.

The phenotype remained sensitive to observation design. Requiring longer fixed-day windows reduced the eligible sample and widened intervals. In the same 52 participants, increasing the daily threshold from 100 to 500 keystrokes attenuated the CV ratio from 1.25 to 1.20, with the latter interval crossing 1.

Parkinson’s disease diagnosis and Impact were self-reported. The one-time Impact category is not equivalent to MDS-UPDRS, Hoehn and Yahr stage, or a validated patient-reported outcome, and its precise question was unavailable. The 73-participant complete-case cohort included only 10 participants in the Severe group, limiting precision for categorical comparisons and more complex models. Missing age may have introduced selection bias. The data lack observed typing-session labels, task context, clinician-rated outcomes, and contemporaneous ON/OFF medication state. The public release contains keyboard-side labels but lacks individual key identities, keyboard-column positions, and observed finger use, preventing key-specific or finger-specific analyses. The 30-min session rule and 50-keystroke fallback blocks approximate within-day dependence. Observation density also affected estimates in the same-sample 100-versus-500-keystroke analysis. This participant-level cross-sectional analysis cannot establish progression tracking or clinical validity.

Day-to-day variability in key-hold duration warrants evaluation alongside average duration in passive fine-motor assessment. Prospective studies should combine passive typing with repeated clinician-rated outcomes and patient-reported function, time-stamped medication exposure, and validation in independent cohorts.

## Data Availability

The Tappy Keystroke Data v1.0.0 analyzed in this study are publicly available from PhysioNet (https://physionet.org/content/tappy/1.0.0/; DOI 10.13026/C2K08D; Adams, 2017b). The public release contains participant metadata, keystroke timing, and keyboard-side labels, but no individual key identities or keyboard-column positions. Summary results are reported in this article and its Supplementary material. The analysis code, unrounded estimates, software environment details, and supporting diagnostic outputs are available from the corresponding author, FY (yangfei@301hospital.com.cn), upon reasonable request.
