# Supplementary material for: Day-to-day variability in naturalistic key-hold duration is associated with self-reported Parkinson’s disease impact
Source: Front Aging Neurosci. 2026 Oct 30;18:1982964. doi: 10.3389/fnagi.2026.1982964 (PMC13759486; doi:10.3389/fnagi.2026.1982964)
Supplement: Supplementary file 1 [file Table_1.docx]

# Supplementary Note 1. Released definition and coding of self-reported PD Impact

The PhysioNet Tappy v1.0.0 data dictionary defines Impact as Parkinson's disease severity or impact on daily life and lists Mild, Medium, and Severe as self-reported options. The release does not provide the exact question wording, response instructions, administration timing, or validation evidence. In the ordinal trend model these categories were coded 1, 2, and 3; the categorical parameterization treated them as nominal levels with Mild as the reference and did not assume equal spacing.

# Supplementary Table 1. Exploratory observation-window truncation analyses

| **Restriction** | **PD n** | **Ratio** | **95% confidence interval** | **P value** |
| --- | --- | --- | --- | --- |
| First 14 calendar days | 62 | 1.399 | 1.069-1.829 | 0.01432 |
| First 30 calendar days | 69 | 1.335 | 1.071-1.663 | 0.01010 |
| First 60 calendar days | 71 | 1.359 | 1.133-1.630 | 0.0009238 |
| First 90 calendar days | 72 | 1.324 | 1.123-1.560 | 0.0008110 |
| First 120 calendar days | 73 | 1.317 | 1.127-1.540 | 0.0005301 |
| Exactly first 5 valid days; >=5 available | 73 | 1.538 | 1.228-1.927 | 0.0001804 |
| Exactly first 10 valid days; >=10 available | 59 | 1.404 | 1.084-1.818 | 0.01019 |
| Exactly first 20 valid days; >=20 available | 37 | 1.359 | 0.971-1.902 | 0.07408 |
| Exactly first 30 valid days; >=30 available | 32 | 1.257 | 0.896-1.765 | 0.18546 |

Calendar-window analyses required at least five valid typing days within each interval. Fixed-day analyses required at least N valid days and used exactly the first N days. Each analysis refitted the primary covariate model. Ratios are exponentiated ordinal-trend coefficients from log-linear models with HC3 heteroskedasticity-consistent standard errors; P values are two-sided. The 20- and 30-day fixed-window intervals crossed 1 as the eligible samples fell to 37 and 32 participants. PD, Parkinson's disease.

# Supplementary Table 2. Exploratory leave-one-calendar-month-out analyses

| **Month omitted** | **PD n** | **Ratio** | **95% confidence interval** | **P value** |
| --- | --- | --- | --- | --- |
| 2016-06 | 73 | 1.324 | 1.135-1.545 | 0.000366 |
| 2016-07 | 72 | 1.289 | 1.107-1.502 | 0.001076 |
| 2016-08 | 71 | 1.327 | 1.134-1.554 | 0.0004339 |
| 2016-09 | 65 | 1.377 | 1.121-1.692 | 0.00233 |
| 2016-10 | 72 | 1.345 | 1.154-1.567 | 0.0001456 |
| 2016-11 | 72 | 1.349 | 1.150-1.583 | 0.0002415 |
| 2016-12 | 73 | 1.330 | 1.133-1.561 | 0.0004803 |
| 2017-01 | 70 | 1.314 | 1.123-1.539 | 0.0006821 |
| 2017-02 | 57 | 1.343 | 1.102-1.638 | 0.003534 |
| 2017-03 | 73 | 1.317 | 1.130-1.535 | 0.0004194 |

Participant-level phenotypes were recomputed after removing each calendar month in turn and refitting the primary model. Ratios are exponentiated Impact coefficients from the complete primary model with HC3 heteroskedasticity-consistent standard errors; P values are two-sided. PD, Parkinson's disease.

# Supplementary Table 3. Secondary phenotype and exploratory sensitivity analyses

| Analysis | n | Ratio | 95% confidence interval | P value |
| --- | --- | --- | --- | --- |
| Raw within-person SD, no mean adjustment | 73 | 1.387 | 1.092-1.761 | 0.00726 |
| RMSSD per Impact category | 73 | 1.363 | 1.154-1.609 | 0.000258 |
| RMSSD, no mean adjustment | 73 | 1.431 | 1.114-1.840 | 0.00510 |
| Consecutive-day RMSSD, >=1 pair | 72 | 1.400 | 1.124-1.744 | 0.00267 |
| Consecutive-day RMSSD, >=2 pairs | 68 | 1.330 | 1.085-1.632 | 0.00616 |
| Consecutive-day RMSSD, >=5 pairs | 57 | 1.238 | 1.001-1.531 | 0.0490 |
| 50/50 hand-standardized CV | 73 | 1.326 | 1.148-1.531 | 0.000121 |
| 50/50 hand-standardized SD | 73 | 1.374 | 1.101-1.714 | 0.00485 |
| 50/50 hand-standardized RMSSD | 73 | 1.366 | 1.170-1.595 | 0.0000823 |
| Mean hold duration per Impact category | 73 | 1.034 | 0.935-1.144 | 0.515 |
| Clinical-characteristic sensitivity: medication and tremor covariates | 73 | 1.288 | 1.067-1.555 | 0.0085 |
| Quadratic curvature | 73 | 0.943 | 0.719-1.236 | 0.672 |
| Bootstrap day-to-day CV | 73 | 1.323 | 1.147-1.528 | - |
| Bootstrap RMSSD | 73 | 1.362 | 1.170-1.589 | - |
| Age omitted | 87 | 1.308 | 1.142-1.499 | 0.000109 |
| Estimated age 50-80 years | 68 | 1.341 | 1.152-1.561 | 0.000150 |
| Additional observation-span adjustment | 73 | 1.302 | 1.107-1.533 | 0.00148 |
| At least 10 valid days | 59 | 1.259 | 1.059-1.497 | 0.00924 |
| At least 200 keystrokes/day | 64 | 1.333 | 1.100-1.616 | 0.00342 |
| At least 200 keystrokes/day and 10 days | 51 | 1.249 | 1.028-1.519 | 0.0253 |
| Data-density sensitivity: at least 500 keystrokes/day | 52 | 1.204 | 0.973-1.490 | 0.0877 |
| Leave one participant out | 73 fits | 1.279-1.377 | All lower bounds >1 | <=0.00125 |

Bootstrap rows report median ratios and percentile confidence intervals from the raw-data rebuild. For the leave-one-participant-out analysis, the ratio column gives the range of point estimates and the P-value column gives the largest P value. Model-based P values are two-sided. The raw SD analysis omits participant mean hold duration. Hand-standardized analyses average the daily means for L- and R-labeled keyboard sides with equal weights, using days with at least 25 retained keystrokes per side and at least five eligible days per participant. These labels represent keyboard regions, not observed hand or finger use. The medication/tremor model additionally adjusts for reported medication use and tremor; contemporaneous medication state was unavailable. The 500-keystroke threshold in this table changes both phenotype construction and cohort membership; a same-sample 100-versus-500 comparison is reported in Supplementary Table 7. CV, coefficient of variation; RMSSD, root mean square of successive differences; SD, standard deviation.

# Supplementary Table 4. Primary categorical parameterization and denominator-independent analyses

| **Analysis** | **n** | **Effect** | **95% confidence interval** | **P value** |
| --- | --- | --- | --- | --- |
| CV: Impact overall (2-df robust Wald) | 73 | Wald statistic 13.18 | - | 0.00137 |
| CV: Medium versus Mild | 73 | Ratio 1.384 | 1.094-1.750 | 0.00671 |
| CV: Severe versus Mild | 73 | Ratio 1.704 | 1.182-2.455 | 0.00426 |
| Raw SD: Impact overall (2-df robust Wald) | 73 | Wald statistic 9.34 | - | 0.00939 |
| Raw SD: Medium versus Mild | 73 | Ratio 1.461 | 1.100-1.941 | 0.00877 |
| Raw SD: Severe versus Mild | 73 | Ratio 1.857 | 1.023-3.372 | 0.04185 |

CV models adjust for age, sex, valid typing days, daily typing volume, and participant mean hold duration. Raw SD models adjust for age, sex, valid typing days, and daily typing volume but omit participant mean hold duration. Overall P values are joint robust Wald tests. CV, coefficient of variation; SD, standard deviation.

# Supplementary Table 5. Exploratory assessment of complete-case selection among participants with known Impact

| **Variable** | **Included n** | **Included median** | **Age-missing n** | **Age-missing median** | **P value** |
| --- | --- | --- | --- | --- | --- |
| Valid typing days | 73 | 21.0 | 14 | 36.5 | 0.422 |
| Median daily keystrokes | 73 | 529 | 14 | 552.5 | 0.777 |
| Observation span, days | 73 | 47.0 | 14 | 47.0 | 0.917 |
| Mean hold duration, ms | 73 | 120.85 | 14 | 130.89 | 0.995 |
| Day-to-day CV | 73 | 0.0643 | 14 | 0.0692 | 0.831 |
| Within-person SD, ms | 73 | 7.80 | 14 | 7.00 | 0.665 |
| RMSSD, ms | 73 | 10.08 | 14 | 9.33 | 0.995 |
| Impact distribution | 73 | - | 14 | - | 0.482 |

Continuous variables were compared descriptively using two-sided Mann-Whitney U tests, and Impact distributions were compared using a chi-square test. No large descriptive differences were observed, but the age-missing group included only 14 participants; non-significant P values do not establish equivalence or ignorable missingness.

# Supplementary Table 6. Post hoc disease-duration and participant-day location-scale analyses

| **Analysis** | **Sample** | **Effect** | **95% confidence interval** | **Inference** |
| --- | --- | --- | --- | --- |
| CV + disease duration | 72 | Ratio 1.341 | 1.144-1.572 | HC3; P=0.000303 |
| Raw SD + disease duration | 72 | Ratio 1.403 | 1.099-1.791 | HC3; P=0.00650 |
| RMSSD + disease duration | 72 | Ratio 1.457 | 1.128-1.881 | HC3; P=0.00394 |
| Location-scale, no sampling-variance term | 72; 3,193 days | Residual SD ratio 1.533 | 1.129-1.806 | Participant-cluster bootstrap (B=2,000) |
| Conventional ME-aware location-scale | 72; 3,193 days | Residual SD ratio 1.579 | 1.134-1.905 | Participant-cluster bootstrap (B=2,000) |
| Conventional ME-aware location-scale | 72; 3,193 days | Residual SD ratio 1.579 | 1.164-2.169 | Mammen cluster wild bootstrap (B=2,000) |
| Session/block-aware location-scale | 72; 3,193 days | Residual SD ratio 1.623 | 1.195-1.976 | Participant-cluster bootstrap (B=2,000) |
| Session/block-aware location-scale | 72; 3,193 days | Residual SD ratio 1.623 | 1.143-2.268 | Mammen cluster wild bootstrap (B=2,000) |
| Disease duration in scale submodel: no sampling term | 72; 3,193 days | Residual SD ratio 0.997 per +5 years | 0.893-1.123 | Participant-cluster bootstrap (B=2,000) |
| Disease duration in scale submodel: conventional ME | 72; 3,193 days | Residual SD ratio 1.003 per +5 years | 0.873-1.135 | Participant-cluster bootstrap (B=2,000) |
| Disease duration in scale submodel: session/block ME | 72; 3,193 days | Residual SD ratio 1.047 per +5 years | 0.894-1.190 | Participant-cluster bootstrap (B=2,000) |

Approximate disease duration was the calendar year of each participant's observation midpoint minus DiagnosisYear, truncated at zero. Participant-day models used log daily mean hold duration with a Gaussian participant random intercept. Location and scale submodels included Impact, age, sex, approximate disease duration, log(valid typing days), and log(median daily typing volume). Models were estimated by marginal maximum likelihood with L-BFGS-B (ftol=10^-10; gtol=10^-7; maxls=40), bounded variance parameters, and 15 estimated parameters. The conventional measurement-error model used the delta-method finite-keystroke sampling variance; the session/block-aware model used an intercept-only cluster-sandwich variance for sessions separated by gaps longer than 30 minutes, with consecutive 50-keystroke fallback blocks on single-session days. Confidence intervals used 2,000 participant-cluster bootstrap replicates; measurement-error-aware models also used 2,000 Mammen participant-cluster wild-bootstrap replicates. Fixed seeds were used, failed fits were retried, and no reported bootstrap replicate failed.

## Supplementary Table 7. Filtering, robust daily-summary, and same-sample sensitivity analyses

| **Analysis** | **n** | **Ratio** | **95% confidence interval** | **P value** |
| --- | --- | --- | --- | --- |
| Primary timing filter: all three measures >0 to ≤800 ms | 73 | 1.324 | 1.135-1.545 | 0.000363 |
| All three timing measures >0 to ≤1,000 ms | 73 | 1.324 | 1.135-1.546 | 0.000372 |
| All three timing measures >0 to ≤1,500 ms | 73 | 1.324 | 1.134-1.546 | 0.000375 |
| Hold duration only >0 to ≤800 ms | 73 | 1.324 | 1.135-1.545 | 0.000363 |
| Daily-median CV | 73 | 1.298 | 1.083-1.556 | 0.00475 |
| Daily-median RMSSD | 73 | 1.349 | 1.115-1.631 | 0.00209 |
| Daily 10% trimmed-mean CV | 73 | 1.319 | 1.123-1.549 | 0.000729 |
| Same 52 participants: >=100 keystrokes/day | 52 | 1.247 | 1.036-1.501 | 0.0196 |
| Same 52 participants: >=500 keystrokes/day | 52 | 1.204 | 0.973-1.490 | 0.0877 |

All ratios are exponentiated ordinal-trend coefficients per one-category increase in self-reported PD Impact. Models use the primary covariate set and HC3 standard errors. The same-sample rows fix the cohort to the 52 complete cases meeting the 500-keystroke/day definition, then recompute CV using each threshold. The >0 to ≤800-ms rule retained 8,999,669 events; the 1,000-ms and 1,500-ms rules retained 8,999,847 and 9,000,034 events, respectively. CV, coefficient of variation; RMSSD, root mean square of successive differences.

## Supplementary Table 8. Location-scale implementation and convergence audit

| **Location-scale model** | **Residual SD ratio** | **Cluster-bootstrap 95% CI** | **Wild-bootstrap 95% CI** | **Failed / 2,000** |
| --- | --- | --- | --- | --- |
| No sampling term | 1.533 | 1.129-1.806 | Not applied | 0 |
| Delta-method ME | 1.579 | 1.134-1.905 | 1.164-2.169 | 0 / 0 |
| Session/block ME | 1.623 | 1.195-1.976 | 1.143-2.268 | 0 / 0 |

All models used 72 participants, 3,193 participant-days, 15 parameters, marginal maximum likelihood, a Gaussian random intercept, and L-BFGS-B optimization. Full-sample negative log-likelihood values were -3443.326, -3475.930, and -3634.559 for the no-sampling, conventional, and session/block-aware models, respectively. Maximum absolute gradients were 0.0128, 0.00878, and 0.00714. Of 5,490 baseline participant-days, 4,934 contained at least two detected sessions (median 4); 556 used consecutive 50-keystroke fallback blocks (median 7). The median session/block-aware log-scale sampling variance was 2.26 times the conventional delta-method value. CI, confidence interval; ME, measurement error; SD, standard deviation.

# Supplementary Table 9. Post hoc inter-key variability analyses in the fixed primary cohort

| **Interval** | **Analysis or contrast** | **Ratio** | **95% CI** | **P value** | **Holm P** |
| --- | --- | --- | --- | --- | --- |
| Inter-key latency | Ordinal trend, per Impact category | 1.20 | 0.99-1.46 | 0.062 | 0.124 |
| Inter-key latency | Medium versus Mild | 1.07 | 0.81-1.41 | 0.624 | - |
| Inter-key latency | Severe versus Mild | 1.57 | 1.01-2.44 | 0.044 | - |
| Inter-key latency | Categorical overall (2 df) | - | - | 0.132 | 0.132 |
| Flight time | Ordinal trend, per Impact category | 1.18 | 1.01-1.38 | 0.039 | 0.118 |
| Flight time | Medium versus Mild | 1.02 | 0.79-1.33 | 0.860 | - |
| Flight time | Severe versus Mild | 1.54 | 1.13-2.10 | 0.007 | - |
| Flight time | Categorical overall (2 df) | - | - | 0.016 | 0.065 |

All analyses used the fixed primary cohort of 73 participants (33 Mild, 30 Medium, 10 Severe self-reported PD Impact). The outcomes were CVs across daily mean inter-key latency or flight time, calculated from the primary retained-event sample (all three timing measures >0 and ≤800 ms; at least 100 retained keystrokes per day and five valid days). Log-CV models used OLS with HC3 standard errors and adjusted for age, sex, log(valid typing days), log(median daily keystroke count), and log(participant mean of the corresponding interval). Holm adjustment covered the two ordinal-trend tests and two categorical omnibus tests. Medium-versus-Mild and Severe-versus-Mild contrast P values and 95% CIs are unadjusted. P values are two-sided. CV, coefficient of variation.

Supplementary Figure 1. Leave-one-calendar-month-out stability analysis. Day-level records from each calendar month were removed in turn, participant-level phenotypes were recomputed, and the primary adjusted model was refitted. Points show adjusted ratios per one-category increase in self-reported PD Impact; error bars show 95% confidence intervals based on HC3 heteroskedasticity-consistent standard errors. The dotted line is the primary full-dataset estimate (1.324), and the dashed line is the null ratio (1). Across ten omission analyses, estimates ranged from 1.289 to 1.377, and all P values were less than 0.004.
